# Exacerbation of Thymoma-Associated Myasthenia Gravis Following Efgartigimod Treatment Related to Anti-acetylcholine Receptor Antibody Overshoot: A Report of Two Cases

**DOI:** 10.7759/cureus.50692

**Published:** 2023-12-17

**Authors:** Kentaro Kawama, Yoko Warabi, Kota Bokuda, Hideki Kimura, Kazushi Takahashi

**Affiliations:** 1 Department of Neurology, Tokyo Metropolitan Neurological Hospital, Tokyo, JPN

**Keywords:** plasma exchange therapy, myasthenia gravis crisis, malignant thymoma, extended thymectomy, neonatal fc receptor, rebound, antibody overshoot, anti-acetylcholine receptor antibody, efgartigimod, myasthenia gravis (mg)

## Abstract

Myasthenia gravis (MG), a chronic, autoimmune disease affecting the neuromuscular junction, arises from various autoantibodies, including those against the acetylcholine receptor (AChR). Recently, efgartigimod, a human IgG1 antibody Fc fragment engineered to reduce the pathogenic IgG autoantibody level, was developed as a treatment for MG. However, the long-term effects of the treatment are still unclear. The present report describes two novel cases of thymoma-associated MG exacerbation following efgartigimod treatment related to anti-AChR antibody overshoot. Both cases shared certain characteristics, including anti-AChR antibody positivity and post-thymectomy status. After a few cycles of efgartigimod treatment, their MG deteriorated, and their anti-AChR antibody titer exceeded the level before efgartigimod therapy. Prior studies show that anti-AChR antibody titer does not correlate with the disease severity of MG. However, previous studies have reported antibody overshoot following plasma exchange, which, like efgartigimod, reduces the level of plasma IgG and autoantibodies. Thus, MG exacerbation with anti-AChR antibody overshoot may be an adverse effect of both efgartigimod and plasma exchange. When clinical symptoms in patients with thymoma-associated MG receiving efgartigimod deteriorate despite low IgG, assessing the anti-AChR antibody level can be important for reconsidering the treatment strategy.

## Introduction

Myasthenia gravis (MG), a chronic, autoimmune disease affecting the neuromuscular junction, arises from various autoantibodies, including those against the acetylcholine receptor (AChR) and muscle-specific tyrosine kinase [1]. Treatment of MG involves the use of corticosteroids, immunosuppressants, such as azathioprine and tacrolimus, plasma exchange, and intravenous immunoglobulin (IVIg). Recently, efgartigimod, a human IgG1 antibody Fc fragment engineered to reduce the pathogenic IgG autoantibody level, was developed as a treatment for MG [2,3]. Efgartigimod is an antagonist of the neonatal Fc receptor, which plays a key role in prolonging the half-life of IgG by salvaging it from lysosomal degradation and recycling it back into the circulation [4]. Efgartigimod induces IgG degeneration by binding the neonatal Fc receptor, thereby decreasing serum IgG. A randomized control study of 84 patients receiving efgartigimod confirmed the safety and efficacy of the treatment for MG [3]. However, because the study period was limited to 26 weeks, the long-term effects of the treatment are still unclear. Moreover, the study did not include patients with MG with malignant thymoma.

Plasma exchange, which removes proteins, including pathogenic IgG autoantibodies, from the plasma, is used to manage MG exacerbation. However, a previous study reported MG exacerbation associated with anti-AchR antibody overshoot following plasma exchange [5]. Efgartigimod in effect removes IgG from the blood, thus performing a function similar to that of plasma exchange. For this reason, MG exacerbation with anti-AChR antibody overshoot may be an adverse effect of efgartigimod. However, to the best of our knowledge, this phenomenon has not been reported previously in patients receiving this drug. The present report describes two patients with thymoma-associated MG exacerbation following efgartigimod treatment related to anti-AChR antibody overshoot.

## Case presentation

Case 1

**Figure 1:**
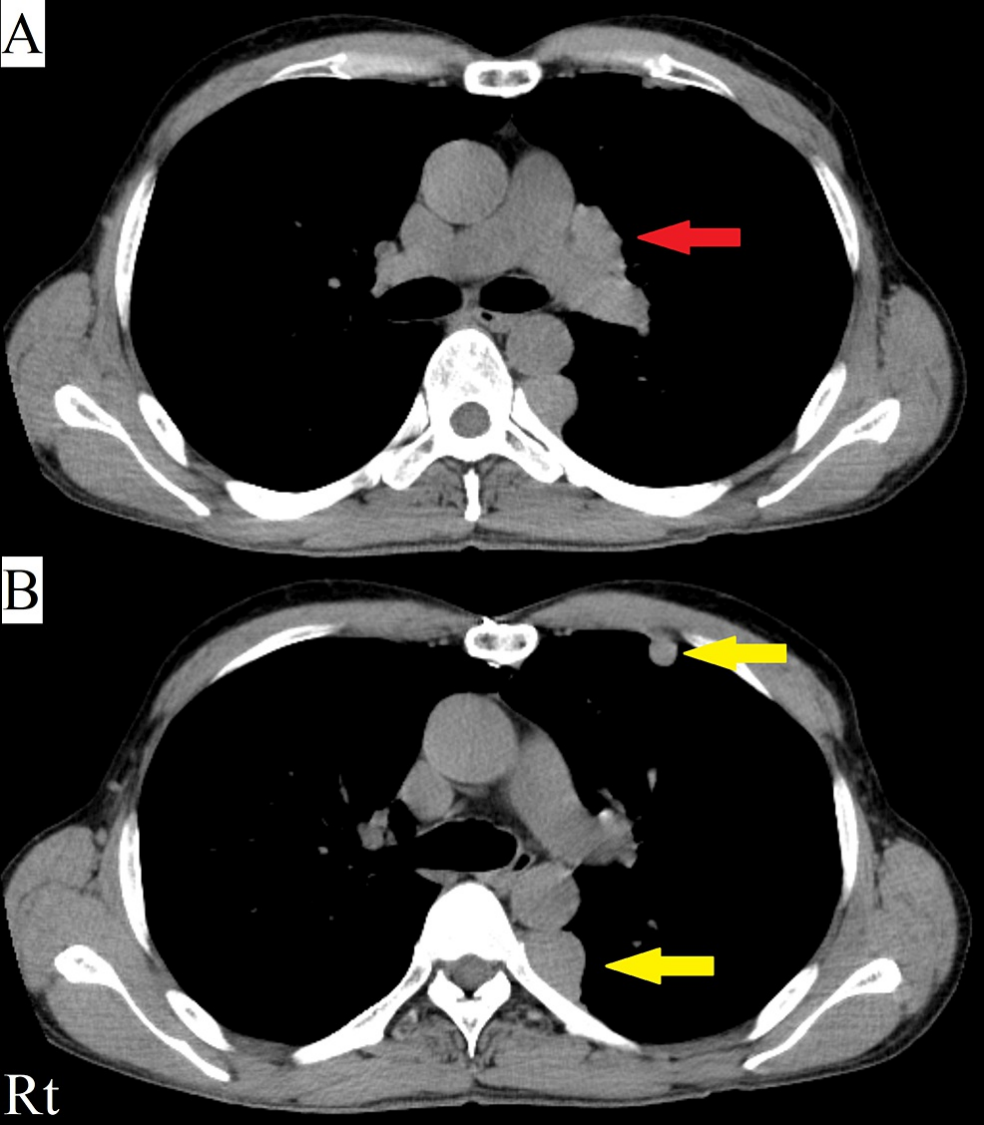
Transverse sections on CT (soft tissue window setting) demonstrating metastatic thymoma in the patient in Case 1. (A) Invasive thymoma metastasis to the mediastinal region (red arrow). (B) Invasive thymoma metastasis to the lateral pleura (yellow arrows). CT, computed tomography

A 52-year-old male patient with anti-AChR antibody-positive generalized MG was admitted three months previously for a sudden deterioration of his condition. His MG first appeared 14 years previously and was marked by dysphagia, ptosis, and upper and lower extremity weakness. Invasive thymoma was also diagnosed concurrently. The initial thymoma excision was performed 14 years previously and was followed by another excision seven years later. However, thymoma metastases continued to be found in the mediastinal region (Figure 1A) and lateral pleura (Figure 1B). His symptoms were managed with oral prednisolone 10 mg/day, oral cyclosporine 250 mg/day, and irregular administration of IVIg for flares. Although his anti-AChR antibody titer fluctuated between 50 and 100 nmol/L over the previous two years, he maintained his activities of daily living and continued to work as a truck driver.

**Figure 2:**
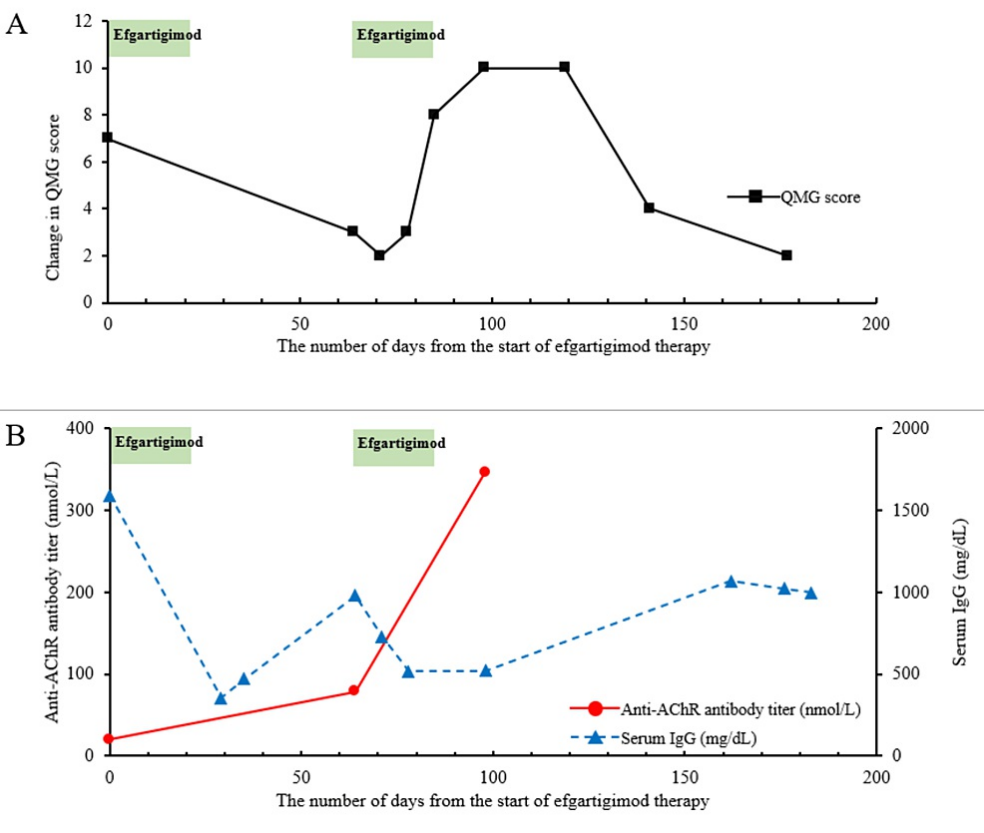
Change in QMG score, anti-AChR antibody titer, and serum IgG level in the patient in Case 1 following efgartigimod treatment. The horizontal axis represents the number of days after the start of efgartigimod therapy. The green bar indicates the duration between the first and last efgartigimod administrations. (A) The vertical axis represents the QMG score (■). (B) The left vertical axis indicates the anti-AChR antibody titer (nmol/L) (●), and the right vertical axis indicates the serum IgG level (mg/dL) (▲). QMG, quantitative myasthenia gravis; AChR, acetylcholine receptor

On admission for a myasthenia crisis, he presented with dysphagia and respiratory failure, which required mechanical ventilation and a tracheostomy. The myasthenia crisis was class Ⅴ according to the Myasthenia Gravis Foundation of America (MGFA) clinical classification. The patient received IVIg and intravenous methylprednisolone (IVMP) as a fast-acting treatment. However, his respiratory symptoms persisted. Following these treatments, plasma exchange was administered four times. During mechanical ventilation, pneumothorax developed, requiring thoracic drain placement. The pneumothorax improved, and the patient was weaned off the ventilator. Efgartigimod was administered to manage the MG symptoms. After the first treatment cycle, the MG symptoms improved, allowing him to walk 50 m without resting. Six weeks after completing the first cycle, a second cycle was initiated based on the findings of a previous randomized controlled trial despite the patient’s having achieved good symptom control [3]. Two weeks after the start of the second cycle, a dropped head developed, and the patient’s quantitative myasthenia gravis (QMG) score rapidly elevated, indicating a rapid worsening of his MG symptoms (Figure 2A). Following the eighth infusion of efgartigimod, dyspnea developed. Two weeks later, he was admitted for mechanical ventilation. IVIg and IVMP were administered as fast-acting treatments; thereafter, the patient was weaned off mechanical ventilation. His anti-AChR antibody titer rapidly increased to 346.2 nmol/L while his IgG decreased to 519 mg/dL (Figure 2B).

**Figure 3:**
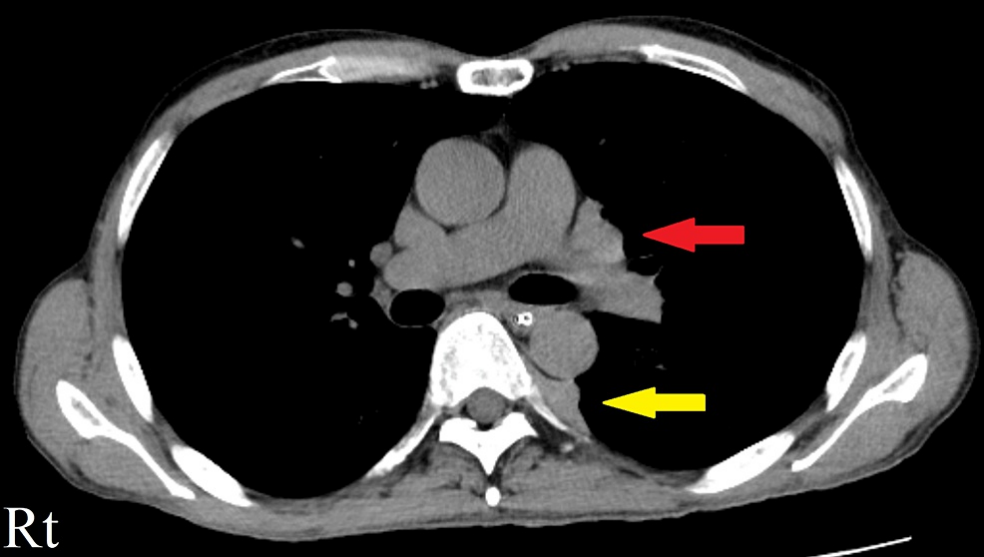
Transverse sections on CT (soft tissue window setting) demonstrating the metastatic thymoma in the patient in Case 1. A slight reduction in the size of the invasive thymoma metastasis in the mediastinal region (red arrow) and lateral pleura (yellow arrow) can be seen. CT, computed tomography

In contrast to the rise in the anti-AChR antibody titer, chest computed tomography (CT) demonstrated a slight reduction in the size of the invasive thymoma (Figure 3). Consequently, efgartigimod was discontinued. Eculizumab was administered to manage his MG symptoms and improved his symptoms and QMG score.

Case 2

A 53-year-old female patient with a ten-year history of anti-AChR antibody-positive generalized MG was admitted for efgartigimod treatment. The initial symptom of MG was ptosis of the left eye. The patient had undergone a thymectomy eight years previously, and a pathological analysis of a surgical specimen found thymoma type 1B. Four years previously, mild dysphagia, classified as class Ⅱb in the MGFA clinical classification, developed. Her symptoms were managed with oral prednisolone 2 mg/day, oral tacrolimus 3 mg/day, and IVIg every three months. Her anti-AChR antibody titer remained stable during the treatment, fluctuating between 2 to 3 nmol/L over the past two years. However, glaucoma and cataracts developed as a result of long-term prednisolone therapy, and her daily activity was restricted by the need for periodic hospitalization for IVIg. Efgartigimod was introduced into the treatment regimen to reduce her need for hospitalization.

**Figure 4:**
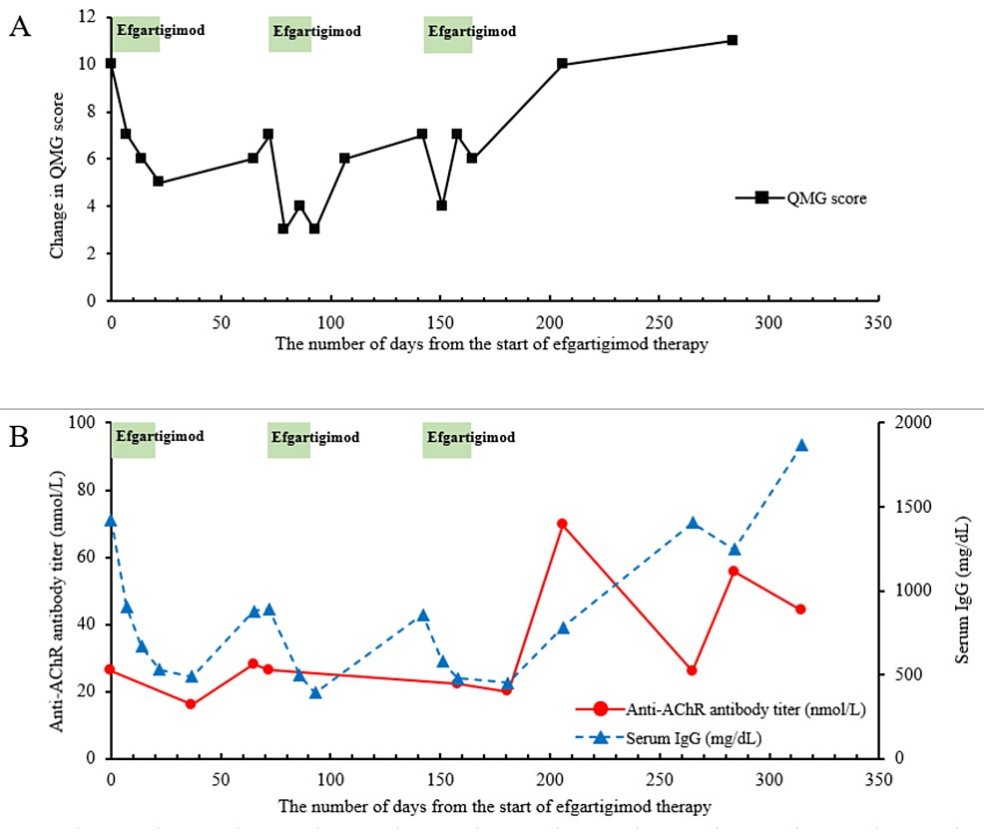
Change in QMG score, anti-AChR antibody titer, and serum IgG level in the patient in Case 2 following efgartigimod treatment. The horizontal axis denotes the number of days from the start of efgartigimod therapy. The green bar indicates the duration between the first and last efgartigimod administrations. (A) The vertical axis represents the QMG score (■). (B) The left vertical axis indicates the anti-AChR antibody titer (nmol/L) (●), and the right vertical axis indicates the serum IgG level (mg/dL) (▲). QMG, quantitative myasthenia gravis; AChR, acetylcholine receptor

The patient received efgartigimod and experienced only minor adverse effects, such as mild hot flashes. Seven days after the first efgartigimod infusion, her QMG score improved by three points and continued to improve until the end of the first cycle (Figure 4A). Seven weeks after completing the first cycle, a second cycle of efgartigimod following a two-point decline in her QMG score resulted in considerable improvement. Seven weeks later, the third cycle was initiated. Her symptoms temporarily improved but returned to their previous severity shortly thereafter. Five weeks later, her diplopia and dysphagia worsened. Her anti-AChR antibody titer rose rapidly to 69.5 nmol/L while her IgG remained low, at 783 mg/dL (Figure 4B), indicating that the effect of efgartigimod was becoming attenuated. Efgartigimod was discontinued, and IVIg was administered as a fast-acting treatment. After that, IVIg was administered every three months. However, her anti-AChR antibody titer was higher than it had been a year earlier, and her symptoms deteriorated slightly.

## Discussion

To the best of our knowledge, the present study is the first to report two cases of thymoma-associated MG exacerbation following efgartigimod treatment related to anti-AChR antibody overshoot. Both patients had the same characteristics, including positivity for the anti-AChR antibody and a history of thymectomy for thymoma.

Efgartigimod is a human IgG1 antibody Fc fragment that increases IgG degeneration by inhibiting its recycling [3]. Plasma exchange and efgartigimod are similar in that they reduce the plasma IgG and autoantibody levels. Ching et al. reported elevated anti-AChR antibodies in patients with MG following a plasma exchange [5]. Four of five patients in their study had anti-AChR antibody overshoot after a plasma exchange despite showing clinical improvement and consequently required more intense immunotherapy. The pathogenesis of anti-AChR antibody overshoot is thought to lie in an increase in B-cells in the peripheral blood lymphocyte population, activation of memory B-cells, and a reduction in antibody feedback inhibition due to a transient reduction in antibodies during plasma exchange [6,7]. A previous study of pemphigus vulgaris reported an overshoot of the anti-Dsg1 and anti-Dsg3 antibodies following a plasma exchange, which occurred within the first two weeks after a plasma exchange possibly owing to the production of autoantibodies by pathogenic B lymphocytes [8]. A similar mechanism may have been at work in the present cases of anti-AChR antibody overshoot following efgartigimod treatment.

Although a randomized control study of efgartigimod did not include patients with MG with malignant thymoma, there is an article describing a favorable course with efgartigimod use of four MG cases with thymoma complications [3,9]. However, the patient in Case 1 in the present study resulted in anti-AChR antibody overshoot following efgartigimod treatment. He had previously experienced a severe myasthenia crisis and had an invasive thymoma metastasis, which might have produced anti-AChR antibodies in excess [10]. Metastatic thymoma remnants might continue to produce anti-AChR antibodies, but preclinical studies have suggested that efgartigimod failed to inhibit IgG production [11]. Although the metastatic thymoma was not analyzed pathologically after the myasthenia crisis in Case 1, the metastasis might have induced an anti-AChR antibody overshoot. On the other hand, eculizumab inhibits the downstream effects of antibodies by blocking the cleavage of complement C5 even while IgG continues to be produced [12-14]. We therefore administered eculizumab to Case 1 in the hope that it would inhibit the downstream effects of antibodies, which resulted in improvement of symptoms. The efficacy and adverse events of efgartigimod in thymoma-associated MG are still under-reported and require careful future findings.

The anti-AChR antibody overshoot associated with efgartigimod in Case 2 was unexpected. Before efgartigimod therapy, the patient’s thymoma had been excised, and her anti-AChR antibody titer was relatively stable, thanks to IVIg administration every three months. Nonetheless, an anti-AChR antibody overshoot occurred, and her anti-AChR antibody titer increased even after efgartigimod was discontinued. As of yet, no clear strategy has been devised for her continuing treatment. Although prior studies show that anti-AChR antibody titer does not correlate with disease severity of MG, when thymoma-associated MG shows signs of deterioration following efgartigimod therapy despite the presence of a low IgG level, measuring the anti-AChR antibody level can be an important first step in reconsidering the treatment strategy. In addition, it is advisable to evaluate the disease status of the thymoma to see if the tumor has recurred.

The present study has several limitations. First, it focused solely on two cases with a relatively short follow-up period. Thus, several aspects, such as the frequency of the anti-AChR antibody overshoot, long-term disease course after the anti-AChR antibody overshoot caused by efgartigimod, and duration of the fluctuation in the anti-AChR antibody level, remain unclear.

## Conclusions

The present study reported two cases of generalized, anti-AChR antibody-positive MG in patients who had undergone a thymectomy for thymoma. After a few cycles of efgartigimod treatment, their MG deteriorated, and their anti-AChR antibody titer exceeded the level before efgartigimod therapy.

Previous studies have reported antibody overshoot following a plasma exchange. Plasma exchange and efgartigimod are similar in that they reduce plasma IgG and autoantibody levels. Thus, the exacerbation of MG with anti-AChR antibody overshoot may be an adverse effect of efgartigimod as it is of plasma exchange. When thymoma-associated MG shows signs of deterioration following efgartigimod therapy despite the presence of a low IgG level, measuring the anti-AChR antibody level can be an important first step in reconsidering the treatment strategy.
